# Oxygen embolism after hydrogen peroxide irrigation during hip arthroscopy: a case report

**DOI:** 10.1186/s12891-020-3081-3

**Published:** 2020-01-30

**Authors:** Zhengwu Peng, Hui Li, Ziqin Cao, Wenchao Zhang, Hongxin Li, Ruping Dai, Lei Liu, Xinzhan Mao, Daniel M. George, Tianlong Huang

**Affiliations:** 1 Department of Orthopaedics, the Second Xiangya Hospital, Central South University, No.139 Middle Renmin Road, Changsha, 410011 Hunan China; 2 Department of Orthopaedics and Trauma, Royal Adelaide Hospital, Adelaide, Australia

**Keywords:** Hydrogen peroxide, Hip arthroscopy, Oxygen embolism, Pyogenic arthritis

## Abstract

**Background:**

Hydrogen peroxide has been widely used in Orthopaedics including Orthopaedic oncology, trauma and joint surgeries. However, we encountered an oxygen embolism and myoglobinuria after hydrogen peroxide was used to irrigate a septic hip arthroscopically.

**Case presentation:**

A 61-year-old male patient with right hip septic arthritis underwent an arthroscopic hip washout and debridement. During the operation, the surgeon used 100 ml of 3% hydrogen peroxide to irrigate the joint cavity. Two minutes after irrigation, there was a transient decrease in oxygen saturation, heart rate and blood pressure, with significant subcutaneous emphysema around the wound. Concentrated urine was drained out 8 h after operation which resolved the following day. Post-operatively, the patient was managed in the intensive care unit for a pulmonary embolism and discharged without further complications.

**Conclusion:**

Medical staff should be aware of the risk of oxygen embolism and be extremely careful when using hydrogen peroxide in patient care. Oxygen embolism following hydrogen peroxide use is rare, however, once encountered, it may bring serious consequences. Therefore, the use of hydrogen peroxide in closed spaces or arthroscopic procedures should be discontinued.

## Background

Septic arthritis of the hip is when bacteria directly causes joint destruction and loss of function. In the past, surgeons would open the joint capsule, dislocate the femoral head if needed, and then irrigate and debride the joint thoroughly. However, during such an operation, the blood supply from the small concave artery and the ascending branch of medial circumflex femoral artery are easily damaged and serious complications encountered. Hip arthroscopy has increased in popularity since the 1970s, which has brought a new option for the treatment of septic arthritis. There have been reports of septic arthritis of the hip successfully treated via hip arthroscopy with good results [1–3].

Hydrogen peroxide can rapidly decompose and release large amounts of oxygen and water after contact with the catalyst synthesized by organic tissues, such as the haem portion of haemoglobin. It is an effective oxidant, and is also a commonly used bactericide, hemostatic agent and surface wound rinsing agent used in clinical practice. However, there are reported cases of hydrogen peroxide causing oxygen embolism in closed or semi-closed operations [4–10]. When the amount of oxygen entering the blood circulation exceeds the absorptive capacity (40 ml), the possibility of sudden death increases significantly [11].

We report a case of oxygen embolism and myoglobinuria after the use of hydrogen peroxide irrigation during hip arthroscopy for septic arthritis.

## Case presentation

**Fig. 1:**
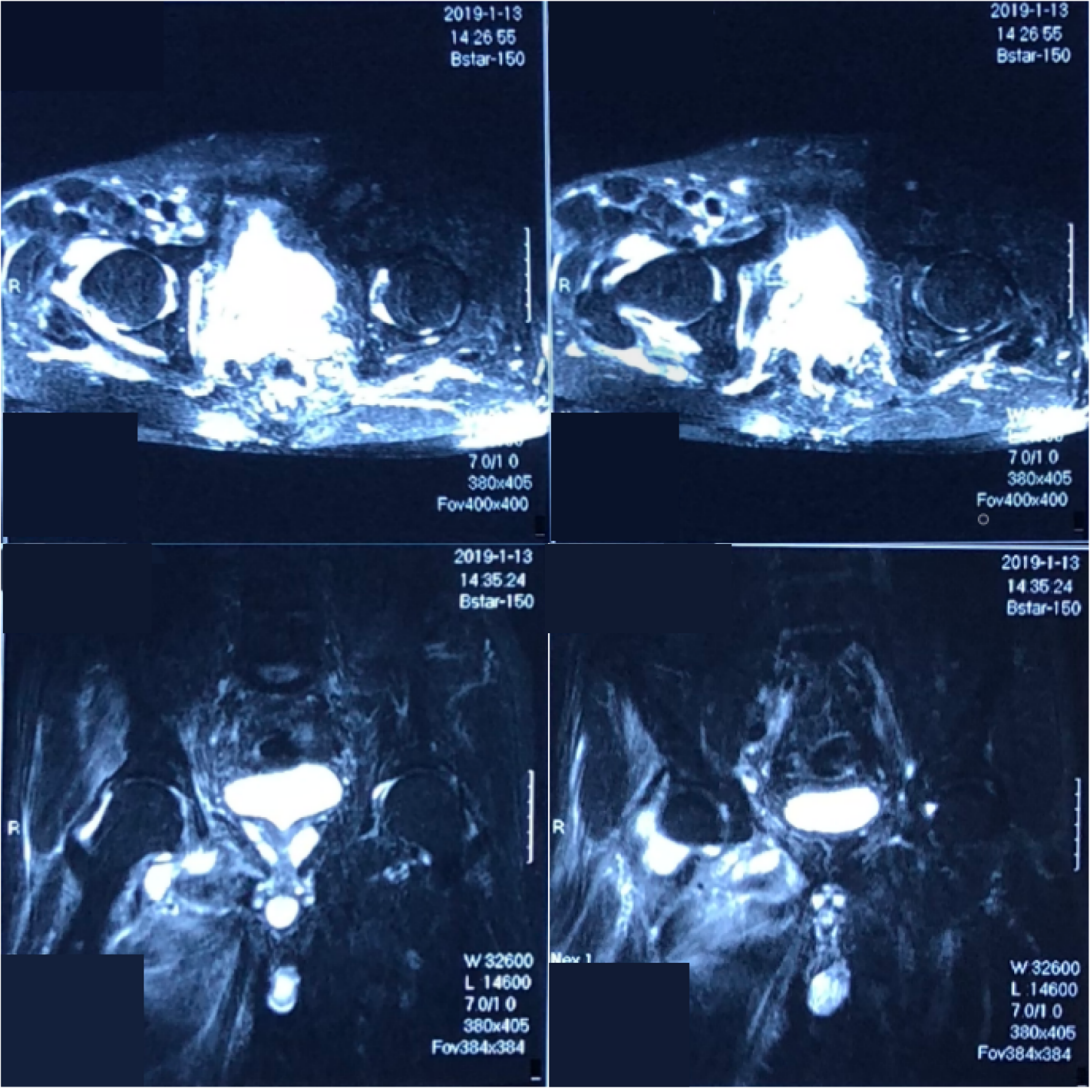
MRI of the right hip demonstrated an effusion and synovitis in the joint

A 61-year-old male was admitted to a rural hospital due to right hip pain for 13 days, aggravated for 3 days. His MRI (Fig. 1) of the right hip demonstrated an effusion and synovitis in the joint. His blood tests demonstrated an elevated white blood cell 15.16 × 10^9^/L, neutrophil 94.6%, C-reactive protein 126.8 mg/L, erythrocyte sedimentation rate 90 mm/h (Table 3-a). After 2 days of intravenous (IV) ceftazidime (2 g BD) he didn’t improve and was transferred to our hospital. The patient has no significant medical history.

On arrival the surgeon performed an ultrasound guided right hip aspiration and 3 ml of pus was aspirated which grew *Staphylococcus aureus* on culture.

The following day the patient underwent a right hip arthroscopy and joint debridement under lumbar plexus block. At the beginning of the anesthesia, the blood pressure was 138/80 mmHg, the heart rate was 92 beats/min, the oxygen saturation (SaO_2_) was 100%, and the end-expiratory CO2 concentration (etCO_2_) was 38 mmHg (Table 1-a).

**Table 1:** Changes of vital signs with time

Time	Blood pressure (mmHg)	heart rate (beats/min)	SaO_2_	etCO_2_ (mmHg)
(a) On anesthesia induction	138/80	92	100%	38
(b) At 12:24	75/50	70	92%	11
(c) At 12:34	90/55	80	93%	17
(d) At 12:50	97/55	80	97%	29
(e) At 13:00 (post op)	90/50	80	95%	\
(f) 40μg/ml norepinephrine 12 ml/h	100/50	\	\	\
(g) 19:00 norepinephrine at 4 ml/h	100/55	80	\	\
(h) At 19:45 (in the ICU)	110/72	96	89%	\
(i) At 09:40 Day 1 post op	131/76	68	97%	\

Table 1 Changes in vital signs over the course of surgery and during the immediate post-operative period. Hydrogen peroxide wash was undertaken arthroscopically at 12:22. (The symbol ‘\’ means that there are not relevant records)

**Fig. 2:**
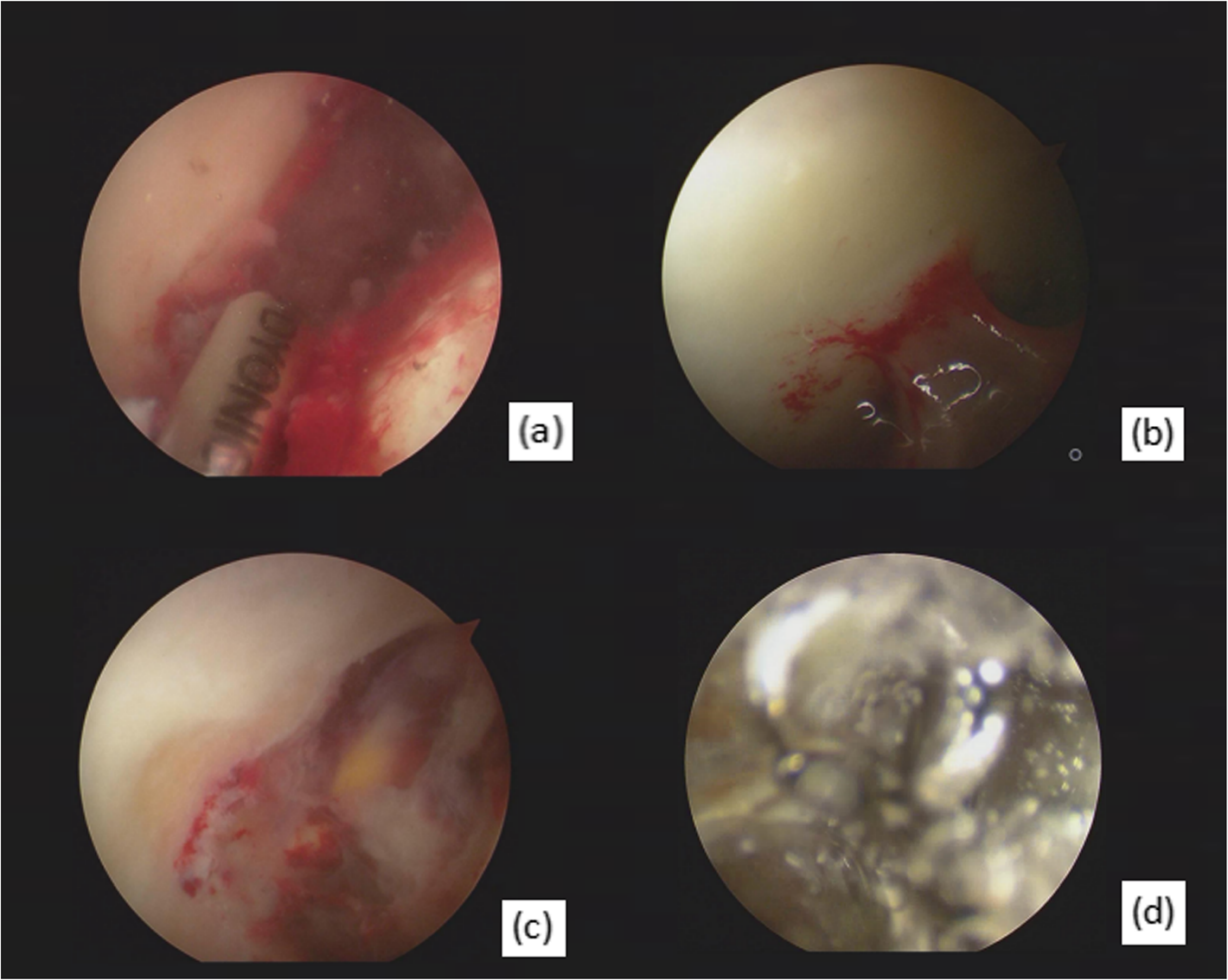
Arthroscopic views revealing pyogenic moss on entry of the joint cavity (**a**), severe synovial and capsular inflammation around the femoral head (**b**), and pus collection in the oval fossa (**c**), The joint cavity was filled with bubbles during hydrogen peroxide rinsing (**d**)

Ceftazidime (2 g IV) was given 30 min prior to induction. Controlled hypotension was performed during the operation, and the blood pressure was maintained at 110/60 mmHg. After satisfactory anesthesia, the patient was positioned supine in traction on the operation table and sterilized routinely. Under bilateral traction, a guide needle was inserted using the lateral approach. The articular space was stretched up to 1 cm. After the guide needle entered the articular cavity, the arthroscope was placed in the direction of the guide needle. Guided by the scope the auxiliary anterolateral portal was established and the anterior articular capsule was cut transversely to expose the acetabulum and femoral head. During the operation, a large number of abscesses were found in the articular cavity and concentrated in the acetabular fossa (Fig. 2). There was extensive synovitis around the articular capsule (Fig. 2). The surgeon debrided the hip joint thoroughly, then rinsed the joint cavity with 100 ml of 3% hydrogen peroxide for 2 min and 10 s, the joint cavity was filled with bubbles. During this process there is no outflow channel maintaining a high-pressure state. No antibiotics were added to the 3% hydrogen peroxide.

There was a transient decrease in oxygen saturation at 12:24, the heart rate slowed down and the blood pressure decreased after 1 min. Left radial artery manometry demonstrated a blood pressure of 70/50 mmHg, heart rate 70 beats/min, with a drop in SaO_2_ 92% and etCO_2_ 11 mmHg (Table 1-b). Acute blood gas analysis showed a PH of 7.30, a PCO_2_ of 44 mmHg, and a PO_2_ of 239 mmHg (Table 2-a). Considering the possibility of pulmonary arterial oxygen embolism, 2 mg of dopamine was injected intravenously, all hydrogen peroxide was extracted out under negative pressure and the joint cavity was washed with a large amount of isotonic saline (18 L used in total throughout the operation), then the joint fluid was drained by negative pressure (Table 1-c + d).

**Table 2:** Changes in blood gas analysis with time

Time	PH	PCO_2_ (mmHg)	PO_2_ (mmHg)
(a) At 12:24	7.30	44	239
(b) After extubating	7.33	43	155
(c) At 15:20	7.40	31	94
(d) At 19:00	7.38	29	77

Table 2 Changes in blood gas over the surgical period. Hydrogen peroxide irrigation at 12:22

At 13:00, the operation was completed. After removing the sterile drapes, the surgeons found that there was subcutaneous crepitus in the right lower extremity, from the anterior superior iliac spine to 5 cm above the ankle joint, the reason for no further subcutaneous crepitus was the elastic bandage used to protect the traction area. The patient was transferred to the resuscitation room with tracheal intubation under anesthesia (Table 1-e). The blood pressure continued to fluctuate requiring norepinephrine (Table 1-f). After extubating in the resuscitation room, the patient was conscious and moving all limbs to command; the blood gas improved, however, the blood pressure required ongoing support with norepinephrine (Table 1-g, Table 2-d). He was transferred to ICU at 19:45 (Table 1-h). The circumference of the most swollen part of right thigh (11 cm under the femoral trochanter) was 56 cm, and the circumference of the most swollen part of the calf (5 cm from the tibial tubercle) was 34 cm. The contralateral thigh and calf circumferences were 50 and 31 cm respectively.

At 19:45, the catheter drained out 800 ml of concentrated dark urine. The ECG showed: sinus rhythm and left ventricular hypertrophy, the blood tests demonstrated: creatine kinase 17,473.1 u/L, kinase isoenzymes 345.8 u/L, myoglobin 11,728.5 μg/L, haemoglobin 106 g/L, lactate dehydrogenase 567.4 u/L, potassium 5.14 mmol/L, high sensitivity troponin T 56.58 pg/ml, N-terminal natriuretic peptide precursor 448.6 pg/ml. The urinary occult blood test was strongly positive (Table 3-c/Table 4-a), which was considered a result of the muscle injury caused by the hydrogen peroxide.

**Table 3:** Changes of blood test with time

Time	WBC (× 10^9^/L)	Hb (g/L)	N%	ESR (mm/h)	CRP (mg/L)	urinary OB test
(a) Prior to arrival	15.16	\	94.60	90	126.8	\
(b) On admission	14.65	112	83.40	94	180.00	negative
(c) Day of surgery	22.38	102	95.60	\	\	strongly positive
(d) Day 5 post op	17.43	99	86.60	97	175.7	positive
(e) Day 12 post op	10.39	97	80.80	92	40	negative

Table 3 Changes in inflammatory markers with time. (The symbol ‘\’ means that there are not relevant records. *N* Neutrophil; *Hb* Haemoglobin; *OB* occult blood)

**Table 4:** Changes of serum myocardial enzymogram with time

Time	CK (u/L)	kinase isoenzymes (u/L)	myoglobin (ug/L)	high sensitivity troponin T (pg/ml)	BNP (pg/ml)
(a) Day 0 post op	17,473.1	345.8	11,728.5	56.58	448.6
(b) Day 5 post op	8645.5	86.9	1747.	15.88	725.2
(c) Day 12 post op	63.4	9.2	39.1	20.29	90.9

Table 4 Cardiac enzyme trends immediate post operatively and over the following days. *CK* Creatine Kinase; *BNP* N-terminal natriuretic peptide precursor

At 09:40 on the 1st post-operative day, the patient’s vital signs stabilised (Table 1-i). He was transferred back to the general ward. There was still subcutaneous emphysema from the wound of the right thigh to the middle of the lower leg. The circumference of the right thigh was 59 cm and that of the calf was 34 cm. After discussion, this patient was diagnosed with an oxygen pulmonary embolism; he received piperacillin tazobactam (Tazocin) (4.5 g Q8h) intravenously for the infection.

On the 2nd day post-operatively, the patient’s right thigh circumference was 54 cm, and there was still subcutaneous crepitus. The calf circumference (10 cm under the tibial tubercle) was 32 cm. On the 5th day after operation, the patient’s vital signs were stable. The right thigh circumference was 51 cm, the calf circumference (30 cm under the tibial tubercle) was 30 cm, and the subcutaneous crepitus had resolved. The blood and urine tests were improving but had yet to normalize (Table 3-d/Table 4-b) The surgical specimens and blood cultures demonstrated *Staphylococcus aureus* septicemia. The patient then was treated with Tazocin plus with linezolid (0.6 g Q12h IV).

On the 12th day after operation, the blood and urine tests had recovered substantially (Table 3-e/Table 4-c). The patient was discharged without further complications.

## Discussion and conclusions

In developing countries, compared with other common antiseptic agents, hydrogen peroxide is cheap and easily available. Theoretically, hydrogen peroxide can kill bacterium by destroying DNA [12], oxidizing protein and membrane lipids [13]. It can also effectively prevent the formation of bacterial biofilms [14]. Therefore, it has been widely used in our clinical practice to treat infections.

However, non-negligible risks have been found, especially in Orthopaedics. It has been reported that hydrogen peroxide can cause chondrotoxicity. It can inhibit the metabolism of normal chondrocytes, deplete the adenosine triphosphate, and reduce the synthesis of proteoglycan and hyaluronic acid in cartilage [15–17]. Hydrogen peroxide also can increase vascular endothelial permeability, pass through the cell membranes via the water channels and cause tissue and cell damage [18]. When hydrogen peroxide is used near blood vessels or organs with rich blood supplies, even traces of hydrogen peroxide (> 5 ml) can produce oxygen [19]. If the process continues, 1 ml of hydrogen peroxide can produce 10 ml of oxygen, therefore, it can lead to a disaster if used in a closed cavity [20]. Akuji and Chambers [21] question the evidence supporting the safety of using hydrogen peroxide during surgery. Although air embolism caused by hydrogen peroxide is rare in arthroscopic surgery, serious consequences of air embolism caused by hydrogen peroxide have been reported in other surgical fields, such as fatal ischemic brainstem lesions and pneumocephalus during spinal surgery [4], tension pneumocephalus and oxygen emboli during a high grade glioma surgery [7], portal venous gas after accidental ingestion of concentrated hydrogen peroxide [8], cardiac arrest during arthroplasty [9], and air embolism after irrigation of external fixator pin sites with hydrogen peroxide [10]. Based on all of its potential safety hazards, surgeons have reduced and even stopped the use of hydrogen peroxide in closed cavities.

The use of hydrogen peroxide in our hospital remains commonplace. Despite previously published reports warning against the use of hydrogen peroxide in closed cavities the uptake of new techniques and knowledge lags behind in developing countries.

In conclusion, our practice needs to be updated as evidence and experiences dictate, and hydrogen peroxide should never be used to rinse closed joint cavities.

## Data Availability

The datasets used and/or analysed during the current study are available from the corresponding author on reasonable request.

## Abbreviations

BNP: N-terminal natriuretic peptide precursor
CK: Creatine kinase
CRP: C-reactive protein
ECG: Electrocardiogram
ESR: Erythrocyte sedimentation rate
etCO2: End-expiratory CO2 concentration
LDH: Lactate dehydrogenase
SaO2: Dioxy saturation
WBC: White blood cell
